# Outcomes associated with bacteremia in the setting of methicillin-resistant *Staphylococcus aureus* pneumonia: a retrospective cohort study

**DOI:** 10.1186/s13054-015-1029-z

**Published:** 2015-09-03

**Authors:** Andrew F. Shorr, Marya D. Zilberberg, Scott T. Micek, Marin H. Kollef

**Affiliations:** Department of Pulmonary and Critical Care Medicine, Washington Hospital Center, 110 Irving St NW, Washington, DC 20010 USA; EviMed Research Group, LLC, PO Box 303, Goshen, MA USA; University of Massachusetts School of Public Health and Health Sciences, Amherst, MA USA; St. Louis College of Pharmacy, 4588 Parkview Place, St. Louis, MO 63110 USA; Division of Pulmonary and Critical Care Medicine, Washington University School of Medicine, 660 South Euclid Avenue, Campus Box 8052, St. Louis, MO 63110 USA

## Abstract

**Introduction:**

Methicillin-resistant *Staphylococcus aureus* (MRSA) remains an important pathogen in pneumonia. Bacteremia may secondarily complicate MRSA pneumonia. The epidemiology and outcomes associated with bacteremia in the setting of MRSA pneumonia are unknown. We sought to describe the prevalence of bacteremia in MRSA pneumonia and its impact on hospital mortality and length of stay (LOS).

**Methods:**

We conducted a single-center retrospective cohort study (2008–2013) including adult patients hospitalized with pneumonia caused by MRSA. We defined pneumonia based on clinical criteria and all cases were culture confirmed. MRSA bacteremia was identified based on positive blood cultures. Pneumonia was categorized as either community-onset (CO, occurring at presentation or within 2 days of admission) or hospital-onset (HO, occurring > 2 days after admission). We compared bacteremic and non-bacteremic groups with respect to their demographic and clinical characteristics and outcomes. A logistic regression and a generalized linear model (GLM) were constructed to examine the impact of bacteremia on hospital mortality and post-pneumonia onset LOS, respectively.

**Results:**

Among the 765 patients with MRSA pneumonia (33.1 % CO), 93 (12.2 %) had concurrent bacteremia (37.6 % CO). Patients with bacteremia were similar to non-bacteremic subjects based on demographic and clinical characteristics with the exception of frequency of a hospitalization within prior 180 days (48.4 % bacteremic and 37.7 % non-bacteremic, *p* = 0.047), prevalence of chronic liver disease (17.2 % vs. 9.5 %, *p* = 0.030), and the mean APACHE II score at the onset of pneumonia (17.5 ± 6.0 vs. 16.1 ± 6.0, *p* = 0.045). Both unadjusted mortality (33.7 % vs. 23.8 %, *p* = 0.067) and median post-pneumonia LOS (18.2 vs. 12.2 days, *p* < 0.001) were greater in the bacteremic than the non-bacteremic group. In a logistic regression, bacteremia showed a trend toward an association with increased mortality (odds ratio 1.56, 95 % confidence interval 0.93 to 2.61). Concomitant bacteremia was independently associated with a 10.3-day increase in the post-pneumonia hospital LOS (95 % confidence interval 6.7 to 13.9 days).

**Conclusions:**

Concurrent bacteremia occurred with moderate frequency in the setting of hospitalization with MRSA pneumonia. Although bacteremia did not appear to independently impact mortality, this was likely due to our study’s limited sample size. However, bacteremia complicating MRSA pneumonia added between 1 and 2 weeks to the hospital LOS.

## Introduction

Rates of infection due to methicillin-resistant *Staphylococcus aureus* (MRSA) have been falling over the last 5 years in both the United States and in Europe [1]. Despite this trend, MRSA remains an important cause of pneumonia and is associated with substantial morbidity and mortality. Specifically, MRSA has been implicated as a pathogen in healthcare-associated (HCAP), hospital-acquired (HAP), and ventilator-associated (VAP) pneumonia [2]. In VAP in the US, for example, MRSA represents the second most common bacterial etiology for this infection [3]. More importantly, crude in-hospital mortality rates in these various MRSA pulmonary infections range from 10 to 30 % [3, 4]. Furthermore, in the US the community-associated strain of MRSA has been reported to be occasionally a cause of community-acquired pneumonia (CAP) and to lead to severe necrotizing infections [5]. In Europe, although MRSA rates have declined rapidly, MRSA pneumonia continues to result in poor outcomes, and several European experts have proposed MRSA pneumonia guidelines to address this syndrome [6, 7].

Significant predictors of survival in MRSA pneumonia include the timeliness of antibiotic therapy, severity of illness at time of infection onset, and chronic underlying conditions [8, 9]. Less certain is the importance of concurrent bacteremia in MRSA pneumonia. In skin and skin structure infections caused by MRSA, secondary bacteremia appears to occur in up to 10 % of patents, but has no impact on mortality [8, 10]. With respect to MRSA pneumonia, little is known about the prevalence of bacteremia complicating this infection, as few reports have analyzed this systematically. Those analyses that have addressed bacteremia in MRSA pneumonia have, generally, been small [11, 12]. Additionally, no information exists regarding whether and how concurrent bacteremia in MRSA pneumonia affects hospital length of stay (LOS), a major determinant of healthcare costs.

In order to clarify these issues, we conducted a retrospective analysis of all patients with MRSA pneumonia treated at a large, tertiary-care hospital. Specifically, we sought to determine the prevalence of concurrent bacteremia in MRSA pneumonia. Moreover, we aimed to describe the impact of bacteremia on both hospital mortality and hospital LOS.

## Methods

### Study overview

We retrospectively evaluated all subjects with MRSA pneumonia admitted to a single institution (Barnes-Jewish Hospital, St. Louis, MO, USA) between December 2008 and December 2013. The study only included adult patients (aged > 18 years) admitted through the emergency department. We excluded those persons transferred directly to the hospital from other institutions. We also excluded patients with polymicrobial respiratory infections. This project was approved by the Barnes-Jewish Hospital institutional review board, and there was no requirement for informed consent given our retrospective design.

Pneumonia was identified based on traditional signs and symptoms of chest infection. We further required evidence of an infiltrate on chest imaging (e.g., either chest radiograph or computed tomographic scan). All radiology studies were reviewed by a single investigator (M.H.K.). Cases were initially identified for possible inclusion in the study cohort through a review of an administrative database of all persons with a discharge diagnosis of any type of pneumonia or of sepsis and respiratory failure. These results were cross-referenced with the hospital’s microbiology system to identify all individuals with positive respiratory and blood cultures showing MRSA (as described below). To be included, patients must have had a respiratory culture which grew MRSA along with the appropriate signs and evidence of pneumonia. Subjects with abnormal chest imaging and blood cultures revealing MRSA but in whom respiratory cultures revealed no growth were excluded.

### End points

In hospital, all-cause mortality served as the primary end point. Hospital LOS following the infection onset represented a secondary end point.

### Definitions and variables

Pneumonia was classified as CAP or HCAP if it was diagnosed within 2 days of hospitalization. In contrast, pneumonia was deemed HAP or VAP if it occurred 3 or more days following admission to the hospital and was not present on admission. VAP specifically required the patient to be on mechanical ventilation for at least 24 hours prior to the infection’s onset. We defined pneumonia as due to MRSA if sputum, lower airway, blood, or pleural cultures were positive for MRSA. Patients without a respiratory culture revealing MRSA were excluded. We further ascertained if bacteremia complicated the patient’s pneumonia. We defined bacteremia as present if at least one blood culture was positive for MRSA. Only blood cultures drawn within 48 hours of the onset of pneumonia were reviewed in order to ensure that the pneumonia and bacteremia were concurrent events. As part of institutional protocol, all subjects with suspected pneumonia undergo routine blood culture sampling (Bactec system, Becton Dickson, Franklin Lakes, NJ, USA). We examined a number of covariates, such as demographic variables (age, sex, race), chronic disease burden (as individual comorbidities as well as their global burden as represented by the Charlson score), acute illness severity (Acute Physiology and Chronic Health Evaluation [APACHE] II score, need for mechanical ventilation and/or vasopressors), laboratory, physiological and treatment parameters [13, 14].

### Statistical analyses

Continuous variables were reported as means with standard deviations and as medians with 25^th^ and 75^th^ percentiles. Differences between mean values were tested via the Student’s *t* test, while those between medians were examined using the Mann-Whitney *U* test. Categorical data were summarized as proportions, and the chi-square test or Fisher’s exact test for small samples was used to examine differences between groups. We developed several multiple logistic regression models to identify clinical risk factors for hospital mortality. In the mortality models, all risk factors that were significant at ≤ 0.20 in the univariate analyses, as well as all biologically plausible factors, even if they did not reach this level of significance, were included in the corresponding multivariable analyses. All variables entered into the models were examined for collinearity, and interaction terms were tested. The most parsimonious models were derived using the backward manual elimination method, and the best-fitting model was chosen based on the area under the receiver operating characteristics curve (AUROC or the c-statistic). The model’s calibration was assessed with the Hosmer-Lemeshow goodness-of-fit test. To examine the determinants of bacteremia-attributable LOS, we constructed a generalized linear model (GLM) with log-link to a Gaussian distribution. Model fit was tested using Akaike information criterion (AIC) and Bayesian information criterion (BIC) tests. All tests were two-tailed. Because of sample size considerations, a *p* value < 0.10 was deemed acceptable a priori to represent statistical significance for the mortality outcome. All remaining tests of significance required alpha < 0.05.

All computations were performed in Stata/SE, version 9 (StataCorp, College Station, TX, USA).

## Results

Among the 765 patients with MRSA pneumonia (33.1 % CAP/HCAP), 93 (12.2 %) had concurrent bacteremia. At baseline, patients with bacteremia were similar to non-bacteremic subjects based on demographic and clinical characteristics with the exception of frequency of a hospitalization within the prior 180 days (48.4 % bacteremic and 37.7 % non-bacteremic, *p* = 0.047) and prevalence of chronic liver disease (17.2 % vs. 9.5 %, *p* = 0.030) (Table 1). Reflecting this, the mean Charlson comorbidity scores were 4.4 ± 3.3 and 4.2 ± 3.4 among bacteremic and non-bacteremic patients, respectively, *p* = 0.340. At the onset of pneumonia, while the mean APACHE II score was higher among bacteremic subjects (17.5 ± 6.0 vs. 16.1 ± 6.0, *p* = 0.045), no significant differences were observed between the groups in the rates of either mechanical ventilation or vasopressor requirements (Table 2). The median creatinine clearance (CrCl), however, was lower in the setting of bacteremia than in its absence (48, interquartile range [IQR] 26–67 vs. 56, IQR 33–79, *p* = 0.019). The most common initial antibiotic in both the bacteremic and non-bacteremic subjects was vancomycin, which was administered to nearly 80 % of subjects empirically. The median time to first anti-MRSA antibiotic administration appropriate for suspected pneumonia (i.e., vancomycin or linezolid) in both arms was less than 2 hours (Table 2).

**Table 1:** Baseline characteristics

	Bacteremia +		Bacteremia −		*P* value
	*N*	*%*	*N*	*%*	
	93	12.16 %	672	87.84 %	
Age, yrs					
Mean [SD]	58.4 [14.1]		58.8 [17.8]		
Median (IQR)	58.5 (53.4, 67.6)		60.8 (48.1, 72.3)		0.622
Race					
Caucasian	51	54.84 %	424	63.10 %	0.392
African-American	39	41.94 %	213	31.70 %	
Asian	0	0.00 %	5	0.74 %	
Other	0	0.00 %	6	0.89 %	
Unknown	3	3.23 %	24	3.57 %	
Gender, female	35	37.63 %	290	43.15 %	0.580
BMI					
Mean [SD]	28.6 [9.1]		29.0 [10.1]		
Median (IQR)	27.5 (21.9, 33.5)		26.9 (22.2, 33.3)		0.941
Hospitalization in previous 180 days	45	48.39 %	253	37.65 %	0.047
Comorbidities					
MI	12	12.90 %	74	11.01 %	0.599
CHF	21	22.58 %	140	20.83 %	0.685
PVD	13	13.98 %	82	12.20 %	0.616
CVD	11	11.83 %	74	11.01 %	0.860
Dementia	3	3.23 %	11	1.64 %	0.237
COPD	30	32.26 %	210	31.25 %	0.905
Connective tissue disease	2	2.15 %	22	3.27 %	0.757
PUD	6	6.45 %	24	3.57 %	0.246
CLD	16	17.20 %	64	9.52 %	0.030
DM	25	26.88 %	158	23.51 %	0.517
CKD	20	21.51 %	99	14.73 %	0.095
CA	19	20.43 %	142	21.13 %	1.000
HIV	1	1.08 %	4	0.60 %	0.478
Charlson score					
Mean [SD]	4.4 [3.3]		4.2 [3.4]		
Median (IQR)	4 (2, 6)		3 (1, 6)		0.340

*SD* standard deviation, *IQR* interquartile range, *BMI* body mass index, *MI* myocardial infarction, CHF congestive heart failure, *PVD* peripheral vascular disease, *CVD* cerebrovascular disease, *COPD* chronic obstructive pulmonary disease, *PUD* peptic ulcer disease, *CLD* chronic liver disease, *DM* diabetes mellitus, *CKD* chronic kidney disease, *CA* cancer, *HIV* human immunodeficiency virus

**Table 2:** Pneumonia and treatment characteristics

	Bacteremia +		Bacteremia −		*P* value
	*N*	*%*	*N*	*%*	
	93	12.16 %	672	87.84 %	
*Pneumonia characteristics*					
Pre-pneumonia LOS, days					
Mean [SD]	11.3 [15.0]		8.6 [13.7]		
Median (IQR)	4.7 (1.1, 15.8)		4.6 (0.9, 11)		0.333
ICU prior to pneumonia	15	16.13 %	94	13.99 %	0.634
Pneumonia type					
CAP or HCAP	35	37.63 %	218	32.44 %	0.318
HAP or VAP	58	62.37 %	454	67.56 %	
ICU at onset of pneumonia	66	70.97 %	485	72.17 %	0.806
ICU transfer after pneumonia onset	5	5.38 %	19	2.83 %	0.198
No ICU during pneumonia admission	7	7.53 %	73	10.86 %	0.372
Specimen type					
Bronchial brushing	0	0.00 %	1	0.15 %	0.565
Bronchial washing	8	8.60 %	33	4.91 %	
Bronchoalveolar lavage	7	7.53 %	55	8.18 %	
Sputum	7	7.53 %	73	10.86 %	
Induced sputum	4	4.30 %	23	3.42 %	
Tracheal aspirate	67	72.04 %	487	72.47 %	
Illness severity					
Mechanical ventilation	71	76.34 %	499	74.26 %	0.705
Vasopressors	40	43.01 %	228	33.93 %	0.104
APACHE II (at pneumonia onset)					
Mean [SD]	17.5 [6.0]		16.1 [6.0]		
Median (IQR)	16 (13, 22)		16 (12, 20)		0.045
Day of pneumonia onset peak WBC					
Mean [SD]	15.4 [7.2]		15.3 [12.5]		
Median (IQR)	14.2 (10.5, 21.2)		13.5 (10, 17.9)		0.232
Day of pneumonia onset peak temperature, C					
Mean [SD]	38.1 [1.1]		38.1 [1.0]		
Median (IQR)	38.2 (37.5, 38.9)		38.2 (37.3, 38.8)		0.834
CrCl (at pneumonia onset)					
Mean [SD]	50.78 ± 31.42		58.84 ± 32.44		0.019
Median (IQR)	48 (26, 67)		56 (33, 79)		
CrCl > 50 (at pneumonia onset)	43	46.24 %	379	56.40 %	0.065
*Treatment characteristics*					
Time to first drug, hrs					
Mean [SD]	8.7 [21.7]		17.6 [41.7]		
Median (IQR)	0.1 (0.1, 4.0)		0.1 (0.1, 18.6)		0.007
Drug					
Ceftaroline	6	6.45 %	12	1.79 %	0.015
Daptomycin	13	13.98 %	13	1.93 %	<0.001
Linezolid	47	50.54 %	219	32.59 %	0.001
Vancomycin	83	89.25 %	559	83.18 %	0.174
Time to drug, hrs					
Ceftaroline					
Mean [SD]	582.7 [562.5]		382.1 [653.6]		
Median (IQR)	448.9 (69.6, 1,180.5)		150.2 (16.6, 440.5)		0.426
Daptomycin					
Mean [SD]	172.7 [252.0]		648.7 [874.6]		
Median (IQR)	54.3 (0.1, 187.7)		430.4 (236.7, 580)		0.017
Linezolid					
Mean [SD]	117.3 [171.9]		138.9 [225.8]		
Median (IQR)	46.5 (12.2, 157.7)		67.3 (23.0, 170.8)		0.280
Vancomycin					
Mean [SD]	0.9 [0.3]		0.8 [0.4]		
Median (IQR)	1 (1, 1)		1 (1, 1)		0.110
Number of anti-MRSA drugs used					
0	5	5.38 %	85	12.65 %	<0.001
1	36	38.71 %	386	57.44 %	
2	43	46.24 %	187	27.83 %	
3	9	9.68 %	13	1.93 %	
4	0	0.00 %	1	15.00 %	

*LOS* length of stay, *SD* standard deviation, *IQR* interquartile range, *ICU* intensive care unit, *CAP* community-acquired pneumonia, *HCAP* healthcare-associated pneumonia, *HAP* hospital-acquired pneumonia, *VAP* ventilator-associated pneumonia, *APACHE* Acute Physiology and Chronic Health Evaluation, *WBC* white blood cells, *CrCl* creatinine clearance, *MRSA* methicillin-resistant *Staphylococcus aureus*

**Fig. 1:**
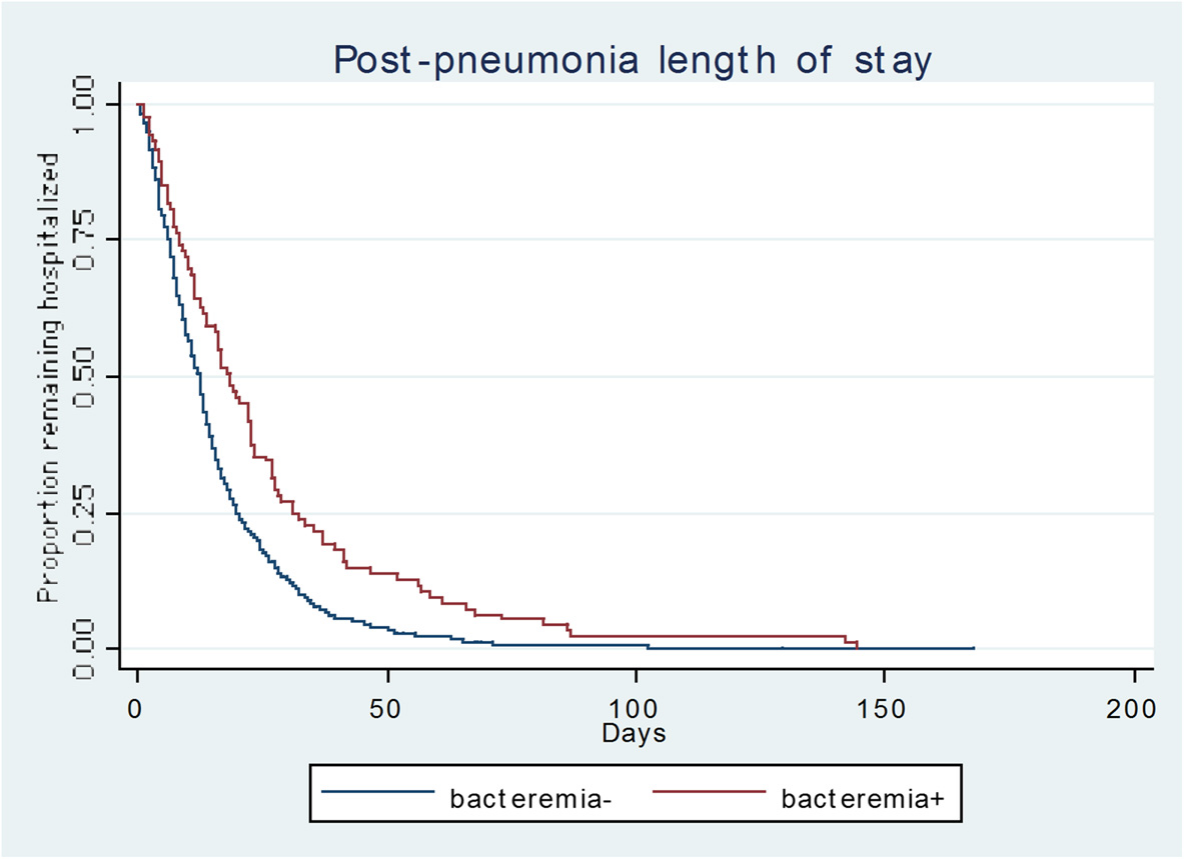
Plot of post-pneumonia onset LOS stratified by bacteremia. *LOS* length of stay

Unadjusted mortality (33.7 % vs. 23.8 %, *p* = 0.067) and median post-pneumonia LOS (18.2 vs. 12.2 days, *p* < 0.001) were greater in the bacteremic than the non-bacteremic group (Table 3, Fig. 1). There were no differences in the discharge destination, with the proportion discharged to home as opposed to a nursing home similar between the groups.

**Table 3:** Unadjusted outcomes

	Bacteremia +		Bacteremia −		*P* value
	*N*	*%*	*N*	*%*	
	93	12.16 %	672	87.84 %	
Post-pneumonia onset LOS, days					
Mean [SD]	25.8 [26.6]		15.8 [16.0]		
Median (IQR)	18.2 (8.1, 31.3)		12.2 (6.1, 19.9)		<0.001
Total LOS, days					
Mean [SD]	37.1 [33.5]		24.4 [22.8]		
Median (IQR)	29.5 (12.8, 46.1)		18.3 (9.9, 31.2)		<0.001
Discharge destination					
Home	6	6.45 %	84	12.50 %	0.067
Rehab	13	13.98 %	97	14.43 %	
LTAC	13	13.98 %	62	9.23 %	
SNF	19	20.43 %	134	19.94 %	
Home with home health services	7	7.53 %	106	15.77 %	
Other^a^	4	4.30 %	31	4.61 %	
Died during hospitalization	31	33.70 %	158	23.80 %	
Died during hospitalization or sent home with hospice care	32	34.41 %	165	24.55 %	0.042

*LOS* length of stay, *SD* standard deviation, *IQR* interquartile range, *LTAC* long-term acute care, *SNF* skilled nursing facility

^a^Category of discharge “other” includes: against medical advice (*n* = 4), home with hospice care (*n* = 8), licensed nursing facility (*n* = 1), psychiatric unit (*n* = 1), federal hospital (*n* = 7), other short-term hospital (*n* = 5), missing (*n* = 9)

In a logistic regression, bacteremia showed a trend toward an association with increased mortality at the alpha level of 0.050, (odds ratio 1.56, 95 % confidence interval [CI] 0.93 to 2.61), and was significantly associated with it at the level of *p* < 0.100 (90 % CI 1.01 to 2.40) (Table 4). Other variables associated with an increased risk of death included measures of disease severity such as the need for mechanical ventilation and the APACHE II score. This model demonstrated a good fit with a Hosmer-Lemeshow *p* = 0.910 and an AUROC of 0.75. Concomitant bacteremia was independently associated with a 10.3-day increase in the attributable post-pneumonia onset hospital LOS (95 % CI 6.7 to 13.9 days, *p* < 0.001) (Table 5). This model for attributable LOS also had a good fit with an AIC of 8.35. Pneumonia type, which was entered into the model, was not retained in the final model as a significant predictor.

**Table 4:** Predictors of mortality

*Parameter*	*OR*	*95 % CI*	*90 % CI*	*P value*
Mechanical ventilation	3.18	1.79 to 5.64	1.96 to 5.14	0.001
Bacteremia	1.56	0.93 to 2.61	1.01 to 2.40	0.091
Vasopressors	1.46	1.01 to 2.12	1.07 to 2.00	0.047
APACHE II (per 1 point)	1.09	1.06 to 1.13	1.06 to 1.12	<0.001
Day of pneumonia onset peak T, C (per 0.1 degree)	0.76	0.63 to 0.92	0.65 to 0.89	0.004
ICU prior to pneumonia	0.29	0.14 to 0.61	0.16 to 0.54	0.001
AUROC = 0.750				
Hosmer-Lemeshow goodness-of-fit *p* = 0.910				
-Factors included but not retained at the *p* value of < 0.1:				
Age, BMI, CAP/HCAP, day of pneumonia onset peak WBC, peak WBC over 7 days after pneumonia onset, CrCl > 50				
-Factors excluded for perfect prediction:				
ICU transfer after pneumonia onset, no ICU during pneumonia admission				
-Factors excluded for collinearity:				
Charlson, pre-pneumonia LOS, ICU at onset of pneumonia				

*OR* odds ratio, *CI* confidence interval, *APACHE* Acute Physiology and Chronic Health Evaluation, *ICU* intensive care unit, *AUROC* area under the receiver operating curve, *BMI* body mass index, *CAP* community-acquired pneumonia, *HCAP* healthcare-associated pneumonia, *WBC* white blood cells, *CrCl* creatinine clearance, *LOS* length of stay

**Table 5:** Attributable hospital length of stay in days

*Parameter*	*Point estimate*	*95 % CI*	*P value*
Bacteremia	10.3	6.7 to 13.9	<0.001
Mechanical ventilation	6.8	3.8 to 9.8	<0.001
Vasopressors	5.6	3.0 to 8.1	<0.001
Time from admission to pneumonia onset (per 1 day)	0.11	0.06 to 0.15	<0.001
Time to anti-MRSA treatment (per 1 hour)	0.03	0.01 to 0.05	0.002
In-hospital mortality	−5.8	−8.4 to −3.2	<0.001

AIC = 8.35

BIC = 156153.9

*CI* confidence interval, *MRSA* methicillin-resistant *Staphylococcus aureus*

## Discussion

This large retrospective analysis indicates that bacteremia complicates nearly one in seven cases of MRSA pneumonia. Although we failed to note an independent impact of secondary bacteremia on hospital mortality, this likely reflects our limited sample size rather that a true absence of an effect. In addition, MRSA bacteremia as a component of MRSA pneumonia adds to the attributable LOS and thus increases hospital costs.

Prior efforts to address the importance of concurrent bacteremia in MRSA pneumonia have been limited. In an analysis of approximately 600 patients with either nosocomial pneumonia or VAP, Magret and coworkers noted that bacteremia occurred in 15 % of subjects, similar to the rate we report. [15] Magret et al., though, included all subjects with either HAP or VAP, irrespective of pathogen [15]. In fact, MRSA accounted for less than 16 % of their study cohort. Nonetheless, they observed that concurrent MRSA bacteremia independently correlated with a higher risk for death. Exploring the issue of the interaction between pneumonia and bloodstream infection from a different perspective, Turnidge et al. analyzed predictors of mortality in a cohort of nearly 2000 patients with *S. aureus* bacteremia, including both those with methicillin-susceptible *S. aureus* and MRSA infection [16]. Persons with concurrent pneumonia and bacteremia only comprised a small aspect of their population (7.8 %) [16]. Pneumonia as the primary infection site, though, appeared to confer a higher risk for death.

In one of the larger efforts to describe variables associated with mortality in MRSA pneumonia, Haque and colleagues analyzed 251 cases of MRSA pneumonia [12]. As others have shown, severity of illness was the main driver of outcomes. However, they failed to even examine concurrent bacteremia as a potential factor affecting eventual survival [12]. Finally, Schreiber et al. specifically explored the potential significance of secondary bacteremia in MRSA pneumonia. They concluded that bacteremia was seen in 20 % of cases of MRSA pneumonia and that bacteremia independently increased the risk for death sixfold. However, this analysis included only 59 subjects and enrolled both those with MRSA and MRSA pneumonia [11]. Reflecting concerns about the seriousness of MRSA as a cause of pneumonia, some have proposed specific MRSA pneumonia treatment paradigms based on the presence or absence of bacteremia, and our results demonstrate the importance of this confounding issue (6.7).

Our study expands on these earlier reports. We confirm a general association between concomitant bacteremia and worse outcomes in MRSA pneumonia. Unlike prior analyses, we evaluated a large cohort of MRSA pneumonia. Furthermore, our cohort was fairly heterogeneous in that it included all cases of MRSA pneumonia, irrespective of pneumonia subtype (e.g., HCAP vs. VAP). This fact helps to establish the generalizability of our findings. The relatively large sample size additionally allows us to better control for a variety of confounders that others could not address as fully. We also establish that concurrent bacteremia adds substantially to hospital LOS. This observation is novel as others have not examined this issue previously. The deleterious impact on LOS is independent of multiple potential confounders. Given that bacteremia remains associated with a greater LOS, our findings suggest that future studies should focus on this cohort of patients in order to determine if some different treatment paradigm might mitigate the negative impact of concurrent bacteremia on LOS. Finally, all patients in this report received timely therapy, reducing the risk that an initial delay in antibiotic treatment confounds our conclusions.

Why might the simultaneous presence of MRSA pneumonia and bacteremia lead to worse patient outcomes? Host factors such as underlying comorbidities could explain some of this connection. For example, patients with bacteremia were more likely to suffer from liver disease than those whose pneumonia was not complicated by bacteremia. However, it seems unlikely that chronic host issues alone account for the excess burden of bacteremia. The absence of any baseline host factors in either the mortality or LOS models indicates that these types of variables likely contribute little to overall outcomes. Alternatively, bacteremia might lead to more severe illness. The presence of bacteria in the blood could theoretically potentiate a cytokine cascade resulting in a more robust systemic inflammatory response. Conversely, bacteremia might simply reflect poor initial source control and a greater organism burden. In either event, the effect of secondary bacteremia on outcomes in MRSA pneumonia likely arises due a complex interaction between the host and the pathogen. However, this relationship is likely more complex than our current tools allow us to elucidate. Haque et al., for instance, analyzed a number of variables related to the bacteria to include its SECmec type, pulsed-field gel electropheresis type, Panton-Valentine leukocidin toxin gene presence and their connection with mortality in MRSA pneumonia [12]. None of these aspects of the culprit MRSA appeared to alter mortality in MRSA pneumonia. Furthermore, the longer LOS may reflect, in part, the choices used to treat patients with concurrent bacteremia. With bacteremia nearly all patients received intravenous glycopeptides. As such, this may have either prolonged hospitalization as outpatient antibiotic therapy was arranged or because outpatient infusion was not an option in select cases. Our findings, hence, may reflect an issue with the choices we have for this serious disease as opposed to some aspect of the bacteremia itself. However, even if this possibility explains some of the difference in LOS between those with and those lacking bacteremia, our data continue to represent a “real world” assessment of the burden of bacteremia in MRSA pneumonia.

Our study has a number of significant limitations. First, its retrospective design opens it to various forms of bias. Specifically, selection bias certainly may have affected our findings. Similarly, ascertainment bias in the identification of bacteremias, due to both the limitations of blood cultures and the frequency with which they are drawn, likely plays a role in our findings. However, we focused on end points such as mortality and LOS, which are not prone to ascertainment or recall bias. We also lacked consistent data on several variables such as the post-infection onset duration of mechanical ventilation. Second, the data derive from a single center and this necessarily limits the generalizability of our findings. In that same vein, the vast majority of our subjects received vancomycin for initial therapy. As such, our results may not be reflective of what one might see if other agents had been employed empirically. Third, despite being a rather large cohort, we nevertheless may have lacked power to adjust for certain confounders that could affect either of our end points. Furthermore, pneumonia itself can represent a difficult diagnosis. To address this concern we utilized precise criteria for the diagnosis of pulmonary infection and had one reviewer examine all chest imaging studies. Fourth, nearly every patient in the study was given vancomycin initially. It remains unclear if vancomycin represents the optimal choice for MRSA pneumonia and therefore our findings might have been different if subjects were treated with other alternative options. With respect to vancomycin, also, we lack data about trough levels. Hence our results may have been skewed by inappropriate dosing even if patients received timely antibiotics. Fifth, given that in some cases patients could have had multiple primary infections occurring concurrently (e.g., a catheter-associated bloodstream infection and a pneumonia) that were misconstrued as one diagnosis, we may have overestimated the frequency of bacteremia complicating MRSA pneumonia. Finally, we could not classify MRSA isolates as to their genotypes (e.g., USA 300 vs. USA 400 strains). Historically in our hospital, USA 300 has been a rare cause of any type of pneumonia. Hence the results we report may not be applicable to settings where the USA 300 strain is far more prevalent.

## Conclusions

In conclusion, concurrent bacteremia in MRSA pneumonia appeared to occur with moderate frequency. Although bacteremia’s impact on mortality did not reach statistical significance at alpha < 0.05, this was likely due to our study’s limited sample size. However, bacteremia complicating MRSA pneumonia added between 1 and 2 weeks to the hospital LOS.

## Key messages

- Concurrent bacteremia occurred in 12 % of intensive care unit (ICU) patients with MRSA pneumonia
- There was a trend toward increased hospital mortality among patients with concurrent bacteremia compared to those without
- Concurrent bacteremia in the setting of MRSA pneumonia was associated with an increase in the hospital LOS of 1–2 weeks

## Abbreviations

AIC: Akaike information criterion
APACHE: Acute Physiology and Chronic Health Evaluation
AUROC: area under the receiver operating curve
BIC: Bayesian information criterion
CAP: community-acquired pneumonia
CI: confidence interval
CO: community-onset
CrCl: creatinine clearance
GLM: generalized linear model
HAP: hospital-acquired pneumonia
HCAP: healthcare-associated pneumonia
HO: hospital-onset
ICU: intensive care unit
IQR: interquartile range
LOS: length of stay
M.H.K.: Marin H. Kollef
MRSA: methicillin-resistant *Staphylococcus aureus*
US: United States
VAP: ventilator-associated pneumonia
